# Adolescent perspectives on the effect of cooperative classroom learning on social networks and health

**DOI:** 10.1371/journal.pone.0328437

**Published:** 2025-07-22

**Authors:** Benjamin P. L. Meza, Lee Simões, Gery Ryan, Marisa Saunders, Kristina Turley, Rebecca N. Dudovitz, Mitchell D. Wong

**Affiliations:** 1 Division of General Internal Medicine and Health Services Research, Department of Medicine, David Geffen School of Medicine, University of California Los Angeles, Los Angeles, California, United States of America; 2 Pasadena High School, Pasadena Unified School District, Pasadena, California, United States of America; 3 Division of Administration of Justice, Technology & Aviation, Glendale Community College, Glendale, California, United States of America; 4 Bernard J. Tyson School of Medicine, Kaiser Permanente, Pasadena, California, United States of America; 5 RAND Corporation, Santa Monica, California, United States of America; 6 Center for Community Schooling, Graduate School of Education and Information Studies, University of California Los Angeles, Los Angeles, California, United States of America; 7 Division of General Pediatrics, Department of Pediatrics and Children’s Discovery and Innovation Institute, David Geffen School of Medicine, University of California Los Angeles, Los Angeles, California, United States of America; The Open University of Israel, ISRAEL

## Abstract

Cooperative learning is a small-group pedagogical technique hypothesized to impact health by altering classroom social dynamics. Using a grounded theory approach, we performed semi-structured interviews with a purposive sample of twelve students at a large urban public high school and explored the effect of cooperative learning on social networks and health. Students emphasized the social risk inherent in group activities and juxtaposed this with the fostering of social-emotional skills, stronger social networks, and protective factors for health. Shy or socially isolated students described significant social anxiety which cooperative learning techniques helped to curtail. These findings highlight the salience of group learning, its effect on adolescent social dynamics, and its potential for influencing health and well-being.

## Introduction

Both observational and experimental evidence finds that school climate impacts a wide swath of important adolescent health and academic outcomes including academic achievement, college matriculation, substance use, mental health, bullying, violent behavior, high risk sex, and other well-being outcomes [1–7]. While there is some consensus that school climate should include multiple overarching domains such as safety, relationships, institutional environment, and school improvement process [8], pedagogical practices and their relationship to adolescent health outcomes has received less attention. Small group learning activities are a form of pedagogy that potentially shape social spaces by connecting adolescents around academic work and require youth to exercise interpersonal skills. Prior research has shown that social networks rapidly change during adolescence and because of the heightened sensitivity to peer influence that occurs concurrently, these networks can have profound effects on adolescent health and behaviors [9–12]. At the school level, the capacity of a system to promote positive, prosocial relationships – its relational capacity – may therefore be a key ingredient for a salubrious school climate. Thus, group learning activities could be a fruitful tool to improve classroom- and school-level relational capacity, school climate and adolescent health via relationship formation and social-emotional skill development. Furthermore, small group learning activities may have cumulative dividends via a virtuous cycle in which positively influential and supportive relationships improve social-emotional skills that then help expand and fortify beneficial relationships [5,13–16].

Cooperative learning is a pedagogical technique that may influence how peers connect to each other in the classroom and thus affect peer social networks and health. Cooperative learning is based on interdependence theory which finds that when a group of individuals work toward a common goal and each group member’s success is contingent on the group achieving that goal, termed *positive interdependence*, individuals are incentivized to have positive interactions with one another [17]. The technique has been incorporated into numerous educational interventions to promote student engagement and achievement including Student Teams-Achievement Divisions, Teams-Games-Tournaments, Jigsaw, Group Investigation, and Learning Together [18]. In such interventions, students are formed into small groups to complete an assignment together. Each of these interventions is unique, i.e., groups may compete against one another as teams, they may have individual members specialize in particular tasks that each contribute to the final product, there may be a peer-coaching or peer-teaching component, but all of these interventions incorporate 1) positive interdependence, 2) individual accountability to perform a task for the group, and 3) feedback and rewards at the individual- and group-levels to incentivize constructive peer interactions [19]. Working cooperatively with peers is associated with a variety of psychological well-being outcomes including emotional maturity, strong personal identity, resilience in the face of adversity, trust and optimism about people, self-confidence, independence, self-esteem, improved social relations, and greater capacity for perspective taking [20]. Interdependence theory further posits that these conditions induce greater openness to being influenced by and influencing others within the group [21].

Social network theory focuses on the structure and composition of the social ties through which social influence is transmitted. Social-structural conditions, such as the classroom environment created by cooperative learning shapes the formation of social networks that subsequently provide opportunities for social influence, support, and engagement leading to downstream health outcomes [22]. An example of this is the process of deviancy training in which academically disengaged youth develop social network connections between one another that lead to inducements to substance use and other risky behaviors [23]. In such contexts, social network effects may account for as much as 50% of the variance in substance use [24,25]. While there is a paucity of literature examining how cooperative environments shape social networks, we hypothesize that positive interactions may foster prosocial networks and thus protect against substance use and other risky behaviors [26,27]. In support of this hypothesis, prior studies have shown that cooperative learning is associated with improved academic outcomes, social-emotional skills, mental health, substance use, bullying, and student well-being [28–37].

Linked Learning, a school reform initiative that has been adopted in school systems in California and nationally, provides a suitable setting to explore this hypothesis [38]. The initiative’s guiding pillars include the inclusion of project-based cooperative learning training and implementation in the form of longitudinal small group projects [39]. As part of these small group projects, students work together toward a common goal, often across multiple class disciplines. Because positive peer interdependence is difficult to achieve, Linked Learning teachers are trained to use specific classroom methods to achieve interdependence, including 1) assessing student performance at the group level such that individuals are incentivized to align with group goals, 2) ensuring individual accountability so no one student carries a disproportionate burden, 3) prioritizing face-to-face peer team-building interactions, 4) identifying and teaching essential social skills, and 5) allowing time for group reflection on the process of collaboration. At the start of Linked Learning implementation and then periodically thereafter, teachers undergo intensive training to develop and practice their curricula under the guidance of experienced trainers. Linked Learning trained teachers guide students through a semester-long, trans-disciplinary group project that integrates skills and all core disciplines. To achieve this intensive experience, students are cohorted such that they can maintain the same peer collaborators across all core classes, and teachers in the core courses can supervise and coordinate the group projects to foster greater peer interdependence. For example, a small group of students could be assigned an architecture-themed project to design a residential apartment in which they calculate energy needs in their science class, formulate a project budget in math, draft an advertising campaign in English, and virtually design the apartment layout in their engineering elective. The project sustains peer collaboration over a longer period of time and more consistently with the same cohort of peers than traditional classroom projects.

Leveraging this practical implementation of Linked Learning, our study objective was to examine student perceptions of mechanisms by which small group classroom projects may influence health, health behaviors, and well-being, including whether relationship formation and social network changes may be important intermediate processes. Understanding whether and how cooperative classroom experiences might ultimately improve adolescent health can identify important leverage points for future interventions that cold support positive academic and health outcomes.

## Materials and methods

### Setting and recruitment

This study purposively sampled participants from a large public high school in Southern California chosen because it had adopted Linked Learning. Following program funding in 2013, the school gave teachers the option of joining one of two Linked Learning pathways which was accompanied by a weekly joint planning period to coordinate transdisciplinary projects with other pathway teachers and a requirement to undergo longitudinal training in project-based and cooperative learning. District Linked Learning coaches, who themselves were trained by a certified third-party organization, conducted the bulk of the teacher training from 2016–2020. During this period, teachers participated in weekly or monthly workshops to develop the school-specific curriculum and have thereafter been provided with intermittent classroom observations and tailored training. The school had four distinct curricular pathways: 1) Creative Arts, Media, and Design; 2) Law and Public Service; 3) App Academy; and 4) a traditional high school curriculum. The first two pathways were Linked Learning certified pathways [40], while the App Academy was targeted to students pursuing careers in information technology. Fewer than 10% of students change pathway after the ninth grade.

This high school, situated within the larger Los Angeles metropolitan area, reflects the broader racial and socioeconomic divide of public schools across the nation, serving primarily low-income students of color [41,42]. In 2021, 62% of students came from low-income households (i.e., received free or reduced lunch), 6% identified as Asian or Filipino, 12% as Black or African American, 59% as Hispanic or Latino, and 19% as white [43]. We recruited a sample of 12 students in the school, half enrolled in a Linked Learning pathway and taught by Linked Learning-trained teachers and half follow a traditional high school curriculum or the App Academy. A purposive sampling technique was chosen to optimize the diversity of perspectives obtained from a small sample including both Linked Learning and traditional pathways, grade, age, and demographics.

We made brief presentations and distributed consent forms in seven classrooms that included all core disciplines, standard and Advanced Placement, as well as both Linked Learning and non-Linked Learning classes. We distributed additional forms to students referred by other students or teachers. Eligible students were: 1) enrolled in grades ten to twelve, 2) able to complete an interview in English or Spanish, and 3) completed the consent process, which included signed parental consent and child assent for students under age 18. Ninth graders were not included in the sample as students had only started high school a few months prior to data collection. Given that each student was expected to have experience with multiple small group projects throughout their high school career, we anticipated needing to recruit 5–10 students in each group (Linked Learning and non-Linked Learning) to be able to describe a diversity of activities and achieve thematic saturation.

### Data collection

We conducted individual interviews on the school campus between September 1 to December 31, 2022 using a semi-structured interview guide (Appendix A). All interviews were conducted where conversation could not be overheard by others such as in an unused classroom, empty outdoor courtyard, or in a counseling office. Interview audio was digitally recorded and professionally transcribed. Each interview lasted 30–60 minutes and was conducted in a single session without interruption. Interviews were conducted until thematic saturation was achieved.

### Interview guide

After an open-ended question about general opinions of school to promote engagement, interviews explored four domains: 1) Student perceptions of the elements of evidence-based cooperative learning integrated into their group work, 2) the influence of cooperative learning practices on student-teacher relationships, 3) the influence of cooperative learning practices on peer relationships, and 4) the influence of cooperative learning practices on mental and behavioral health. The first domain asked about elements of evidence-based cooperative learning including: group assignment, forms of interdependence, face-to-face promotive interactions, individual accountability, interpersonal skills teaching, group processing, and overall attitudes [44]. The second domain asked students to describe how group work influenced their relationships with their teachers, distinguishing between cooperative and non-cooperative group work. The third domain similarly asked students to describe the influence of group work on peer relationships including friendships, classmates, and conflict. Finally, the fourth domain asked students to reflect upon ways in which group work and the relationships they developed through group work influenced their general preferences, behaviors, emotions, and stress, including mental health and risky behaviors. The guide was iteratively reviewed and refined in collaboration with the research team and based on feedback during pilot testing.

### Transcription

Recordings were transcribed verbatim. The main author checked all transcriptions for accuracy. Participants were permitted an opportunity to review transcriptions. No participants elected to do so.

### Data analysis

We used a constructivist grounded theory approach for data analysis [45] using QualCoder 3.2 [46] and Microsoft Excel [47]. The main author completed five stages of analysis (i.e., familiarization, identifying a thematic framework, indexing, charting/rearranging the data, and mapping/interpretation), with the support of Datagain (https://datagainservices.com/about-us/), a commercial transcription and qualitative analysis company, who provided a second transcript reviewer to improve consistency and reliability. Familiarization via immersion in the raw recordings, transcripts, and personal notes occurred following each interview. We conducted a comprehensive review with identification of a thematic framework to date after the third, ninth, and final interviews. Indexing, whereby thematic tags were applied to the textual data was performed as transcripts became available with iterative revisions thereafter. Two reviewers coded and recoded each transcript using a revised coding system. Reviewers discussed all coding questions and conflicts and the research team resolved any remaining conflicting codes between the two reviewers. Reviewers use QualCoder and Microsoft Excel to tag and rearrange the data [46,47]. The research team conducted a final mapping and interpretation using the complete dataset.

One of the original aims of the research was to compare student perspectives of those in Linked Learning to non-Linked Learning pathways with the presumption that the former would experience more frequent and higher quality cooperative learning techniques in small group activities. We quickly found that cooperative learning practices were being used in both groups and differentiating the two would not be feasible using our current methodology. As a result, our analyses aggregated all students and examined cooperative learning techniques regardless of academic pathway.

### Ethics

This study received approval from the Institutional Review Board of the University of California Los Angeles and the partner school district. All participants and their parents received a written information sheet which included a summary of the context and purpose of the study, participant rights (e.g., confidentiality, privacy, informed consent, voluntary participation), and contact information for the research team and the University of California Los Angeles Office of the Human Research Protection Program. Participants over the age of 18 years provided written consent. Minors obtained written consent from at least one parent before providing their own written assent. Consent and assent included permission to record and transcribe interviews as well as publish anonymized results.

### Researcher characteristics

The first author conducted all interviews and performed all analyses. BPLM is a pediatric and internal medicine physician and part of a multidisciplinary research group including physicians, psychologists, and educators. He received training from the Life Course Intervention Research Network and colleagues in the conduct of semi-structured interviewing and qualitative data analysis.

## Results

The sample consisted of twelve students with seven enrolled in a Linked Learning pathway and roughly equal representation from grades 10–12 (Table 1). Participants’ age ranged from 15 to 18 years (mean age 16 years old) and seven participants identified as female with a diverse racial/ethnic representation. Most participants were US born (92%) and spoke English as their first language (75%). One student completed the consent process but moved and was unreachable to complete the interview.

**Table 1:** Participant Demographics (N = 12).

Female	7 (58%)
Male	5 (42%)
Age, mean	16 years
Grade	
10	4 (33%)
11	3 (25%)
12	5 (42%)
Linked Learning	7 (58%)
Race/Ethnicity	
White, non-Hispanic	5 (41%)
Hispanic	3 (25%)
Black or African-American	1 (8%)
Asian or Pacific Islander	2 (17%)
American Indian	1 (8%)
Other	1 (8%)
Don’t know	3 (25%)
US Born	11 (92%)
English as first language	9 (75%)

Four overarching themes emerged: the role of social-emotional skills, social dynamics of collaboration, the built learning environment, and health consequences. Within the first theme, students discussed *relationship skills* and *other social-emotional skills* that mediated how small group cooperative activities influenced social network changes. In the second theme, students elaborated on how collaboration impacted social processes at the following levels: *dyadic*, *small group*, and *network* levels. When asked what effect cooperative learning had on student-teacher relationships, most students described the social environment of the classroom and the role of teachers in cultivating the learning environment. Within this theme, students discussed *social inclusion and exclusion, fairness*, *support*, and *quality of the cooperative learning experience*. In the final theme, *health consequences*, students discussed *physical health*, *behavioral health*, and *mental health*.

One early finding was the widespread use of cooperative learning techniques in both the Linked Learning and traditional high school classrooms. These techniques were in use heterogeneously across cohorts. Of particular note, small group teaching in the App Academy emulated professional software development teams and naturally tended to align well with cooperative learning principles.

### Social-emotional skills

Students shared a variety of perspectives describing how social-emotional skills and cooperative activities enhance one another reciprocally. While comments touched on all five of the Collaborative for Academic, Social, and Emotional Learning (CASEL) domains including social awareness, self-awareness, self-management, and responsible decision making, most comments pertained to the domain of relationship skills [48,49]. Perspectives reflected a range of domain competence including highly proficient communication, leadership, resistance to negative social pressure, and relationship building skills as well as comments that revealed the speaker to have much more limited skill mastery and insight.

#### Relationship skills.

Students’ opinions and attitudes of cooperative projects were deeply influenced by past experiences and a personal assessment of the social risk involved in any such project. To that end, several students expressed a preference for working with close friends to minimize this social risk while also acknowledging that working with non-friend peers had the potential to enhance relationship skills and personal growth.

Leadership, communication skills, and self-advocacy were relationship skills that carried special salience in cooperative activities. Perceived deficits resulted in problematic group experiences and worse “social anxiety” among group members while skill competence was associated with more positive experiences and reduced anxiety. One student (female, traditional curriculum) explained that some disciplines (e.g., science) lend themselves better to group work but, above all, she felt she needed some mastery of the discipline to integrate peers’ points of view and foster a cooperative social dynamic. Other respondents questioned their ability to work effectively with other students and personal leadership skills. Lamenting their own lack of leadership skills, a student (male, Linked Learning) stated that if “nobody is really a leader, then nothing gets done.” The same student described the opposite extreme in which one of his teammates who had a “take charge” personality hindered the contributions of other team members resulting in a ruinous final product. A separate student (female, Linked Learning) with self-described “social anxiety” put it differently. Reflecting on her own lack of self-advocacy, she felt unable to “stick up for” herself and, as a result, her experience in group projects was stressful and less rewarding.

Students described how cooperative activities stimulated a continuum of social relationships characterized in terms of friendship, trust, respect, productivity, and health. Their terminology was almost as variable as the layers of nuance they perceived, ranging from intimate friendships to strictly productivity-based classmate relationships. The influence of cooperative activities on the skills necessary to make and maintain these relationships was similarly nuanced. Some students who assessed their own relationship skills as lacking found cooperative activities helpful for skill development while other students who displayed confidence but demonstrated deficits in active listening or collaborative problem-solving found cooperative classroom activities as difficult or undesirable.

Particularly for students who reported feeling socially isolated, cooperative group projects offered a nurturing environment for refining a range of skills that aide initiation and maintenance of supportive peer relationships. These skills included respectful and effective communication of one’s needs and democratic methods for decision making. Referring to students who find social engagement challenging, one gregarious student (male, Linked Learning) said: “I feel like more social people need to just treat them with as much kindness as they can, make them feel as comfortable as they can, talking, bring up ideas, asking them for their input, stuff like that.” Many of the social skills that students discussed are important for preventing interpersonal conflict, however, several respondents lamented the difficulty they felt managing existing conflict. Some students endorsed seeking help from teachers with variable success.

#### Other social-emotional domains.

Students described scenarios during cooperative activities in which they were socially aware including acknowledging the strengths or challenges of other peers and showing compassion. These experiences were also often connected to moments of self-awareness including identifying personal assets; acknowledging emotions like anger, frustration, or pride; and feeling a sense of purpose in the shared goal. Self-management skills including resilience were mentioned in the context of adapting to the deficits of others in their group or perceived unreasonable expectations of teachers. Finally, group activities engaged students to consider their personal role in a larger group, community, or institutional context. The group context prompted students to reflect on their role within a larger community. Positive group experiences were linked with more expansive interdependent roles while negative experiences tended to elicit more narrow roles. Social-emotional skills were not learned or practiced in isolation but commonly cooccurred during cooperative activities.

### Social dynamics of collaboration

All interviewees recognized the role that peers have on personal habits, emotional states, and behaviors. Close friendships had the most apparent influence, however, students discussed multiple social dynamics that may serve to mold the larger peer social network including factors that strengthen or weaken peer social connections, friendship selection, and social inclusion or exclusion. The following is a description of some of those health-related social dynamics.

#### Dyadic level.

At the dyadic level, students described how they formed new relationships or deepened existing relationships with peers during group activities, but some also noted that stresses inherent in group work could strain or weaken relationships. The relationships that formed ranged from casual acquaintances to trusted classmates to intimate best friends. Students gave examples in which the group activity was sufficient to spark a friendship between previously unacquainted peers: “we were like okay let’s do projects together since you’re like right next to me and then we became good friends after that” (female, Linked Learning). But often, the group project was a link in a series of events that brought students together in friendship. For example, one student (male, Linked Learning) described how he had met one of his best friends during a school project but the friendship only crystalized when they shared a second class together. He stated, “I think because at first we were acquaintances, even after that project but then he switched into my math class and I guess we just started getting closer because we had already met each other, knew each other through that project. It enabled me to talk to him more because I guess I felt more comfortable with him because I had already been through that with him I guess.” The same student noted the challenge of socialization after the isolation of the SARS-CoV-2 pandemic school closures: “Because last year I was way more antisocial than I am now. I had a hard time going up to people and talk to them.” Other factors that reinforced or amplified instances when cooperative projects promoted positive peer relationships included: new or existing friendships with a mutual third student, cooccurring friendship between parents of students, a shared avoidance of peer “drama,” and the evolution of the relationship to include more shared activities (e.g., recreational activities, other group projects).

Although several students reported beginning or expanding their positive social relationships through group projects, some students pointed out that because the activity took place in the classroom, it was unlikely to have implications for socialization outside the classroom. Speaking about group projects generally, one student (male, Linked Learning) said, “I don’t think it necessarily improves on relationships, because like, it’s mostly just a working relationship, but like you always become closer with that person, but it’s, in my experience, you really only become closer during that time where you guys are working”.

Group work in the classroom could strain or perhaps weaken existing relationships, particularly when the work pitted the expectation of loyalty to a friend against responsibilities as a student. In one example, the interviewee (female, Linked Learning) described how her friend was procrastinating on her part of the project which caused a schism in their friendship: “I think a school project can really affect the friendship because if you’re like, hey, you’re not doing this and the other person already feels like it’s personal. […] “Girl, you need to start writing because we need to present this.” And right away I kind of snapped on her […] And that kind of just brought conflict up.” In such situations, students were unlikely to seek aid from teachers to navigate the conflict. Particularly when poor effort from one student was rationale for the second student to also make a poor effort, both friendships and academic performance were harmed. However, under other circumstances, students generated constructive ways of navigating this tension including: 1) compartmentalizing the roles of classmate and friendship (e.g., “right now you’re my classmate. Once we leave this classroom, you are my friend, and we can talk social life and all this. But while we’re in there, it’s like, okay, this is our grade, let’s do this kind of stuff,” female, Linked Learning), 2) rationalizing that different individuals work at different paces, or 3) using humor to buffer conflict.

#### Small group level.

A common theme at the small group level was the importance of a shared goal. As one student (female, Linked Learning) stated, “I feel like even though I wasn’t friends with the people who were in the group, we all worked really well together, because we all wanted to get this done and get a good grade on it.” Positive group experiences were characterized by high group productivity but also a feeling of mutual support and intimacy that may have been unattainable at larger scales: “I think it’s in smaller projects you could open up more to others. Then if its classroom, you wouldn’t say that out loud that, for example, your parents are divorced and stuff like that. You wouldn’t want everyone to know that” (female, non-Linked Learning).

Mutual support and the expectation of mutual support was a strong motivator to work hard and build relationships with peers. In a class characterized by cooperative projects in which assignment completion was dependent on students working together, one student (male, Linked Learning) described his motivation to perform: “Definitely grades. But also I guess maybe just appearing like someone that people can depend on, […] someone that people want to work with.” Students described elements of a virtuous cycle between mutual support and the expectation of such support. Receiving support from peers promoted reciprocation and students saw the fruits of interdependence as greater openness to share ideas, build relationships, and potentially create a better final product.

Yet such harmony did not always occur in group work, and in fact, students described several pitfalls that could demotivate individual students including anonymity, lack of individual accountability, and social isolation or exclusion. One student (female, non-Linked Learning) pointed out that individual work may allow for greater individual attention from the teacher: “when you work on your own, you could kind of work on your own pace and the teacher sees what work you did, and when you’re in a group, you kind of don’t emphasize like what role you took in the group. So it just kind of gives everyone that like feeling like, oh I don’t have to put my full effort to like a group project, the teacher is not gonna know what I did.” And yet, group members did not want to feel as though they were policing their peers: “We tried talking to them. You don’t want to be rude. We can’t be like, ‘Do your work.’ So you’re like, ‘Hey, could you maybe work on your slide, please.’ And he kind of ignored us and didn’t really respond. Another time he’s like, ‘Yeah, I’ll get around to it,’ and obviously, it happened to be right before the presentations, so there wasn’t enough time to practice” (female, Linked Learning). And finally, when a group member felt they were being excluded, either purposefully or because of poor group leadership, the excluded student was sure to lack motivation to contribute and left with a poor opinion of group work. Similarly, in one case of bullying, both the bullied student and the student doing the bullying were perceived to have suffered injury from the experience.

#### Network level.

Students also expounded on the effects of group work within their social networks in two respects. First, students reported relative favoritism for peers who were both socially inclusive and reliably productive. As one student (male, Linked Learning) reported: “but when it comes to people who are students who are I guess more obnoxious than others, or go out of their way to make the teaching harder for the teacher or go out of their way to just make fun of random people that they don’t even know, I don’t know, it just makes me not want to talk to them or cooperate with them in any way.” Both students who reported making friendships as part of group projects as well as those who did not, reported that they would seek out groupmates who were both socially inclusive and academically productive.

Students at the periphery of the network (i.e., having few or no friends) reported a second network phenomenon, which was the social freedom that having at least one good friend conveys. “I think I just became more secure with who I am when I’m with [my friends], I guess, or even when I’m not with them, but knowing that like I have people I can depend on, it makes me feel safe, I guess. I’m not afraid to speak my mind anymore, I just cut people off. Because I’ve had some relationships last year and this year that have been really one sided and weird, so I just dropped them. My good friends gave me the confidence to do that” (male, Linked Learning). Teens that feel socially isolated may feel locked into unhealthy relationships, but a single good friend can provide confidence to shed deleterious relationships.

### The built learning environment

Much like the larger school environment, group work in the classroom can feel to students as a high-stakes game with great social opportunity as well as great risk. One student (female, Linked Learning) encapsulated the challenges inherent in unstructured group work: “There’s like a lot riding on it, because you’re like, ‘Am I doing too much? Am I not doing enough?’ You don’t want people to think you’re lazy, but you also don’t want to take over the whole thing and do everything yourself, like ‘Why isn’t anybody else doing anything?’ And they’re like, ‘Where can I step in?’” Overall, students had much more to say about how cooperative activities in the classroom impacted peer relationships than teacher-student relationships. However, students did attribute the conditions for project success to their teachers.

#### Teacher as architect.

Student perspectives focused on group assignment, fairness, and teacher support. Participants had a myriad of opinions regarding the optimal method for assigning students to small groups and while the methods differed, all students sought to optimize the positive peer social interactions and academic productivity that was described above. For some students, they simply wanted to work with their close friends as much as possible. Students who reported few friends or social anxiety preferred the teacher or a non-peer third party (e.g., random-assignment) make group assignments such that their relative social vulnerability would not be scrutinized.

Group assignments and navigation of social dynamics impacted student perceptions of fairness and support in the classroom. In adoration of one beloved teacher, a student (female, Linked Learning) said: “he has a really good way of communicating with his students, so I think he does these projects because he knows his students and he knows everyone is capable of doing them. Because he’s not the type to put you in the awkward position like other teachers might do, ‘Okay, group project,’ and don’t think about the psychological effects because some kids actually get really bad anxiety from that.” Fairness was challenging when students’ final grades were dependent on working collectively. Students responded positively to teacher accommodations for problematic group dynamics: “we did have conflicts within the group and we just kind of brought it up to her and she kind of helped us by just like understanding like okay, well I won’t knock you off for that person” (female, non-Linked Learning). Other methods of getting student input on the evaluation phase was similarly welcome in the eyes of students: “at the end of each project that I’ve done, we’ve had to do a report on who did what and what grade do you think the other people in the group got and then you evaluate yourself as well” (male, Linked Learning). But when a teacher was not perceived as being sympathetic, a poor grade could symbolize a fracture in the student-teacher relationship despite reassurances to the contrary: “I was kind of upset in my […] teacher, because he didn’t really help us navigate the fact that our groupmate ditched on us. ‘You guys figure it out, and I’m not going to help you guys. You’re still getting this as a bad grade if you don’t pull through though.’ So I was kind of upset about that, because it wasn’t our fault, but other than that, it didn’t really have an effect on- It doesn’t affect my opinion or respect for my teacher” (female, Linked Learning).

Perceived teacher support was discussed as intimately connected to fairness. One student (female, Linked Learning) described, “I had a group project in physics where I specifically told the teacher I did not want to work with the student that was assigned to me […] because he’s not going to do anything.” When the project was not completed to the student’s standards, she was met by callousness from the teacher and she felt betrayed: “Well, it’s a group project and it happens, you guys can redo it for a better grade.“[…] So I think that kind of impacted my relationship with the teacher because it’s like he knew and he still put me with him.” Perceived teacher support was perhaps even more important for students who perceived group work to reduce teacher attention for individual students. Some teachers were perceived to be more involved in helping students navigate social conflict, which students viewed positively.

#### Evidence-based cooperative learning.

As previously mentioned, student reports of the use of evidence-based cooperative learning techniques in the classroom were highly variable. Some group projects included all or most elements of high-fidelity cooperative learning praxis. For example, the computer science pathway routinely emulated software development teams in their group assignments. Given that professional software development is a highly cooperative enterprise, the assignment leant itself easily to many evidence-based practices including interdependence of roles and final reward, group process, and even social skills instruction. As one student (male, non-Linked Learning) described, “but the group project overall, it was really good because everyone was assigned a role like researcher, developer, artistic. So it really did help that we had groups and it was fun as a project overall. Because we did have a lot of time to talk and connect and really show all of our ideas and show that each of us can contribute something to the group project which I really enjoyed. […] So in case someone had something that day, they can’t really finish it that night, that person like, of course, I can help you and just finish it up. The whole team together would succeed.”

The cooperative learning elements that were at times missing were 1) setting up interdependence between the students that went beyond grading the group as a unit (i.e., reward interdependence), such as creating complimentary roles, resources, or developing a group identity; 2) social skills instruction, especially techniques to troubleshoot manage conflict, inclusive decision making, and appreciation of groupmates; and 3) group process, wherein students provide one another encouragement and constructive feedback. One notable exception to the lack of group processing were examples in which students developed their own methods of constructive feedback, generally motivated by existing friendships: “And I’ll give her feedback – she gives me feedback on my essays – and not because she’s my friend, I’m going to tell her like, ‘Oh, your essay is good.’ Like, ‘No, your subsection is missing this, this, and this and that.’” (female, Linked Learning).

### Health consequences

Respondents generally perceived their overall health to be very good and none reported a significant physical illness or substance use disorder. They attributed their current health and health behaviors to a varying combination of individual qualities and social or environmental influences. No students drew a direct connection between cooperative activities in the classroom and their health but nearly all respondents commented on the role of social network influences.

#### Physical health.

Closer friendships but not less intimate relationships such as classmates, were perceived to impart influence over health practices. Speaking for their own personal habits, some students endorsed greater physical activity or attention to healthy eating because of influence from friends: “I started playing a sport for freshman year and then I noticed that my friends started to play a sport as well. […] So I did see that some of the pattern like if your friend does something that more people tend to do it because it’s also scary when you want to start something but you don’t necessarily know where to begin because they’re like ‘what if I’m by myself? What if people judge me?’ and all of that. But when you do already have a friend that’s already doing something, […] I can definitely feel more comfortable in this environment” (male, non-Linked Learning).

#### Substance use and bullying.

Although no student endorsed substance use personally, many students had knowledge of substance using peers. In such descriptions, substance use was described in juxtaposition to academic performance. Respondents stated that their friends were academically oriented and therefore reinforced values that were not compatible with substance use. Many of the same students described how cooperative classroom activities motivated them toward academic achievement and, for some, friendships that formed during cooperative activities were the same that buffered them against risky health behaviors. Overall, students perceived substance use to be both a result of influence as well as homophily (i.e., substance using peers forming friendships between one another more than with non-substance using peers), where homophily around antisocial behaviors like substance use took place outside of school or in unsupervised spaces such as bathrooms.

Insofar as there was a direct relationship between group projects and bullying, one student (male, non-Linked Learning) described himself in terms consistent with bullying: “Every friend group has like that one person like people sometimes target a little bit. […] And we do have that friend which is unfortunate and it’s like I do feel bad for him […] Like mentally I don’t think he’s doing so well […] and I asked him why and he’s like, oh, school work is overflowing on me and he has so many insecurities […] Because although he does not deserve what’s been said, he does act a bit different. So that’s why as a group we make fun of him a little bit because he does say these things, do weird things.” While this bullying was not strictly in the context of group activities, whether group activities could facilitate such bullying deserves further investigation.

#### Mental health.

Several students endorsed personal struggles with mental health or perceived mental health struggles in peers. Foremost was a preoccupation with social acceptance and avoidance of social rejection for which group activities could precipitate either. Furthermore, the relationship between group work and mental health was described as bidirectional: mental health struggles created barriers to full participation in group work and working in groups influenced mental health.

A pretext for all discussions of mental health was the transition from exclusively online classes during the SARS-CoV-2 pandemic to in-person classes. While respondents unanimously preferred in-person instruction, the transition from online to in-person was cited as a stress- and anxiety-producing transition. In some cases, key members of one’s social networks (e.g., best friend) were absent and students felt isolated. In other cases, anticipation or perception of social “awkwardness” provoked anxiety. Group work had the potential to amplify social friction either by pairing students who might otherwise avoid one another or by the mandate of collaboration without guidance on how to navigate social friction. One student (female, Linked Learning) related the outcome of a problematic group pairing over two years earlier on a group project that lacked qualities of evidence-based cooperative learning. Her teacher had dismissed her request to be reassigned, telling her simply to learn how to work with her peers. “And I was like, okay, we do the presentation, and I’m already getting anxiety from this presentation because it’s like, ‘Oh my God, he doesn’t know what he’s doing.’ And like, throughout the classroom, he knew what he was supposed to say but he thought it was funny, like he thought, yeah. Like he was like the kid that would get up there and start laughing when his friends saw him. So I was like, ‘Oh my God.’ So I’m already having anxiety, I presented my part, which was horrible, that was like the worst presentation. I was getting anxiety and I was like -- so yeah.” Here, principles of cooperative learning including promoting individual accountability of group members and structured social skills teaching might have facilitated a more productive and supportive relationship between students as well as promotion of individual resilience in the face of adversity. Instead, the group experience had the opposite effect.

Strong friendships were a potent mitigating factor for anxiety, whether by offering a non-judgmental ear or by showing support through gifts of food served up at vulnerable moments. As a result, some such students preferred to work with friends in cooperative projects because they felt this made the environment more comfortable, however, it was also a shared sentiment that working with non-friends on group projects had benefits, specifically for perspective taking. Teacher empathy and support was another important mitigating factor. Finally, students reported less anxiety when the project had a clear goal in which all group members were invested. Having a shared goal reduced interpersonal friction and increased mutually supportive actions between group members even in the absence of evident friendship formation.

## Discussion

Student perspectives reveal the complex social dynamics of small group cooperative activities in the classroom and downstream health. Under the right conditions, these activities can promote more robust, reciprocally supportive peer networks that empower prosocial and academically productive students while contributing to the relational capacity of the school community. However, creating high quality cooperative environments is a challenging task. Our findings reiterate that students perceive group projects to be as much a social task as an academic one. Students seek guidance and support commensurate to the perceived social risk of the task. The implications of problematic social dynamics in the classroom may be even more hazardous in the post-pandemic period which has been characterized by a period of limited socialization and increased prevalence of psychosocial health issues [50]. Using the lenses of interdependence and social network theories, we identified how students perceive the effects of cooperative learning on peer relationships and downstream health consequences including improved risk factors for physical health, substance use, and mental health. Students reported multiple potential mechanisms between cooperative learning and health including social-emotional skill development, improved peer relationships and social acceptance, academic motivation and engagement, and restructuring of social networks.

Several of the key findings align well with the literature. The finding that elements of cooperative learning were being used in both Linked Learning and non-Linked Learning classrooms is not unexpected. Cooperative learning has a strong evidence base demonstrating improvements in educational outcomes and has been incorporated in many teacher training programs, yet this study’s findings also confirm that cooperative learning is often implemented with low-fidelity and heterogeneously across classrooms [51–53]. Students in this study reiterated that cooperative classroom experiences have the potential to reduce anxiety [54] and increase motivation to overcome difficult tasks [55], however, students also expounded on ways in which certain group activities provoke social anxiety specifically. The duality of reducing and provoking anxiety has not been a focus of existing literature. Our findings could reflect several potential processes including: a new post-pandemic social environment; generational differences as the majority of research into cooperative learning was conducted in student populations prior to the ubiquity of smartphones and social media; specific implementations of group activities that diverged from evidence-based cooperative learning; or an as yet unexplored externality of cooperative learning. Although we cannot form firm conclusions using this study design, respondents suggested that better implementation of some elements of cooperative learning including achieving goal-oriented interdependence, ensuring individual accountability, and teaching social skills for difficult group dynamics may help to mitigate some of the social anxiety associated with cooperative group activities. Such a finding has been reported in implementations of cooperative learning both in-person and online. In these instances, lack of faculty guidance was associated with few student groups achieving positive interdependence, individual accountability, and mutual support [56,57]. Meanwhile, greater guidance and structure resulted in more predictable improvements in behavior and academic performance [56–58]. Some education experts assert that social skills including leadership, decision making, trust building, communication, and conflict management are so fundamental to cooperative learning that any group of students who will be engaging in cooperative learning should first be assessed on these skills and deficiencies addressed prior to any cooperative learning activity [59–61]. Certainly, these interviews suggest that social-emotional skills, particularly interpersonal skills, are important to students and an area for specific attention. Other researchers have observed that students become more independent and the role of teachers evolves as students gain experience with cooperation and with age [19,57]. The findings of this study go further to suggest that while teacher training and skills in conducting cooperative learning predictably elevate academic outcomes, such training and skills also have the potential to improve student health and health behaviors.

The early literature describing cooperative learning demonstrated evidence that cooperative learning may increase friendship formation between students of diverse racial, cultural, and academic achievement backgrounds [26,62,63]. Some limited research has begun to examine how cooperative learning may alter larger peer social networks. On the dyadic level, respondents in this study reported new friendships and working relationships with their peers as a result of cooperative projects. This may be of particular importance for students who see themselves on the social margins, a finding corroborated by literature that finds cooperative learning can reduce social isolation for vulnerable youth [64]. Yet the same students perceived greater risk of social harm when group activities lacked structure. One reason for this social risk may be because high-fidelity cooperative learning promotes empathy and social cohesion that is particularly important for socially marginalized youth [65,66] but unstructured group activities may not. There is much less work examining the network level effects of cooperative learning. The current social network literature supports the general idea that cooperative learning may reduce network hierarchy, clustering, and cliques but the mechanisms are not well delineated [67,68]. We uncovered at least two potential mechanisms contributing to this effect: 1) students who perform well academically and are socially adept are promoted to central positions in the network during cooperative learning and 2) cooperative learning bestows a type of social lubricant allowing youth in unhealthy or marginalizing relationships the ability to shed those relationships for mutually supportive ones. We also found that there is a dynamic interplay between classmate relationships and friendships which remains understudied using quantitative techniques of social network analysis. These network changes are particularly important as we learn how important social dynamics may be for school-level relational capacity and downstream adolescent health. As these respondents reported, peers have wide ranging effects, both directly and indirectly, on adolescent physical health, substance use, bullying, and mental health.

The impacts of the SARS-CoV-2 pandemic add an additional layer of complexity to our analysis. At the time of the interview, students had been back to in-person instruction for more than 1.5–2 years, thus the majority of their reflections focused on post-pandemic experiences. Nevertheless, several students remarked how both school instruction and the school social environment had changed in the post-pandemic era. While the lingering impacts of the pandemic were not a focus of this study, we must consider how to compare pre-pandemic studies with our current findings in two respects: the social environment and pedagogical technique. With the pandemic still in the recent past, much of the post-pandemic literature remains short-term or speculative but there are data suggesting that youth social development may have been impeded during lockdowns resulting in impaired social skills, less stable social networks, and vulnerability to emotional dysregulation [69,70]. Under these conditions, the potential dividends of cooperative learning are enhanced while the conditions for effective classroom management may be more challenging to achieve. Cooperative learning would be expected to improve issues of social-emotional skills, network isolation, and even mental health that were neglected during the pandemic. However, the success of cooperative learning interventions may be blunted if students must first learn the basic social skills needed to work in groups [51]. Furthermore, the teaching profession has changed in some dramatic ways. Greater teacher burnout and turnover along with new teaching modalities including digital tools will reduce potential health benefits of techniques like cooperative learning [71]. On the other hand, many schools have broadened their perception of the role of school and enhanced their capacities for addressing mental health and social circumstances that impact student well-being [72], which could facilitate renewed investment in health promoting pedagogy. Ultimately, the field will need more time and data to determine the developmental trajectories of students and the efficacy of pedagogical techniques like cooperative learning in the post-pandemic era.

### Strengths and limitations

This sample of youth was uniquely situated to examine diverse perspectives of the effects of cooperative learning for several reasons. Foremost, within this single school, students were cohorted into pathways that demonstrated a range of pedagogy from low quality cooperative activities to rigorous, longitudinal, project-based small group work lead by teachers with substantial training in project-based and cooperative learning techniques. Next, Linked Learning cohorting of students permits greater continuity between students across disciplines and years of schooling, likely permitting greater social bonding and network effects that might be diluted out in a sample with fewer opportunities for longitudinal peer interactions. Finally, student respondents self-identified with a diverse set of race/ethnicities, genders, nativity, and ages attending public high school.

This study also had several limitations. First, despite the diverse pedagogic and demographic setting, respondents were sampled from a single public high school in the Los Angeles metropolitan area. The small sample and specific educational context limit our ability to generalize to other settings. As demonstrated in Table 1, the sample was not statistically representative but was purposively sampled to optimize the diversity of perspectives. Further studies should use quantitative study designs and population-based sampling methods to determine the statistical representativeness of these findings. For some of our data, particularly when assessing fidelity of pedagogy to evidence-based cooperative learning, we relied on student report. Especially at young ages, students are likely not aware of all the pedagogical techniques that are being implemented. Future studies should include classroom observation, teacher reports, or the use of validated instruments to assess the presence and quality of key cooperative learning elements. Nevertheless, we were primarily interested in student perspectives, including their perspectives on how group activities were managed and perceived benefits and challenges. Student recall is another potential area for bias. Some students chose to report on activities that happened nearly two years prior to the interview. While we wanted to learn from salient experiences, respondents may not have been able to reliably report important details. Lastly, while individuals may be able to report on social dynamics within the network, they are unlikely to be able to perceive the larger classroom or school network. Therefore, our hypotheses regarding network-level effects must be tested using network-level analyses. Future research should employ sociometric methods to capture whole network data to examine how cooperative learning might modify network structure and how such changes relate to health outcomes.

## Conclusions

While school climate has long been considered an important determinant of adolescent health, pedagogy is not often included in that construct. This qualitative study of the health-related social dynamics of cooperative learning in a non-experimental setting described the potential effect of cooperative learning on health, highlighted many of the potential benefits and challenges, and elucidated mechanisms by which it may improve adolescent health. Teachers who have been trained to think of pedagogy as strictly transmission of knowledge may not see themselves as moderators of youth social dynamics or stewards of relational capacity. Yet mounting evidence suggests that teachers’ pedagogical techniques influence health in important ways and, as professionals, they can be important health-promoting agents when provided the training and support. Additional research is already examining the ways in which teachers effectively collaborate to optimize learning for students with special biopsychosocial needs. Perhaps a more novel implication of this work is for public health practitioners. In practical terms, these findings may help inform school- and community-level interventions to improve adolescent health that synergize with ongoing educational initiatives through the promotion of effective cooperative learning environments. More broadly, as we gain greater understanding of the upstream determinants of health on the life course, we must acknowledge the role of social environments, particularly during periods of development that are distinctly sensitive to social influences, such as adolescence. This study has generated hypotheses around potential mechanisms of effect including social-emotional skill development, improved peer relationships and social acceptance, academic motivation and engagement, and restructuring social networks. Situated in a greater literature on cooperative learning, the findings should suggest to public health practitioners that support of teachers and schools in providing high quality, evidence-based pedagogy may be a novel mechanism for addressing upstream social determinants of health.

## Appendix

### Linked Learning Interview Questions

**Grand Tour Question:**

What is it like to be a student here? Or What’s it like to be in a Linked Learning Program?

**Probes:**

- How it different from other schools?

#### Domain 1: Program fidelity to evidence-based collaborative learning interventions.

Describe for me some of the group activities that you do in your classes.

**Probes:**

- Group assignment: Describe how the group was created and who was in your group. How many people are typically in each group?
- Goal Interdependence: Describe the product that you created for the activity. Was it one product for the whole group or separate products for each student?
- Task Interdependence: Can you walk me through the different steps in the activity and/or toward creating the end product(s)? In what ways did you depend on others in the group to move from one step to the next?
- Face-to-face Promotive Interactions: What parts of the activity did you do together in-person, virtually, and separately? Which mode did you prefer and which mode felt most engaging?
- Role Interdependence and individual accountability: How did each student know their role in the activity? If someone is not doing their part, what happens? What types of things make sure everyone contributes?
- Resource Interdependence: What types of materials and resources did you use to complete the assignment? How did each group member know which materials and resources to use? Did you depend on each other to take advantage of all the materials and resources?
- Identity Interdependence: How would you describe your group identity? How did things like creating a group name, competing with other groups, or feedback from the teacher affect your group identity? Were you proud of your group? Was the group identity a reflection of your personal identity?
- Reward Interdependence: How was the product graded? Did everyone in the group get the same grade? If not, how did the teacher decide which students received higher or lower grades?
- Interpersonal skills: Describe what “people-skills” you practiced to complete the assignment (e.g., completing tasks, communication, decision making, managing conflict, appreciating other group members). What skills did you discuss as a group or as a classroom?
- Group processing: Describe some of the feedback that your group members gave each other to improve your group work or make future group work run more smoothly? At what stage did this happen? Who prompted you to give feedback and who helped the group keep it constructive?
- Attitudes: Overall, what do you like about group activities? What do you not like? What do you think are the most important parts of a group activity? Describe for me your ideal group activity.

#### Domain 2: Effects of collaborative learning on student-teacher relationships.

Describe your relationship with your teachers and how group assignments influence that relationship.

**Probes:**

- Which of your teachers use group activities in the classroom?
- Describe your relationship with each of your teachers.
- What types of things help you and your teacher: 1) communicate better, 2) build trust, 3) build mutual respect?
- What types of things help you to stay motivated on your school work and which of your teachers are good at motivating you?
- What types of things turn you off from school? What types of things strain your relationship with your teacher?
- What do your teachers do that make you more confident in your ability to get things done and manage all your schoolwork?

#### Domain 2: Effects of collaborative learning on peer social networks.

Some people think group activities in the classroom and out of the classroom are an important way to get to know your peers and build your friendship network. In your experience, how do group activities in the classroom and out of the classroom affect who form friendships with whom and what types of friendships they are?

**Probes:**

- Who do you hang out with? How did you start hanging out with them?
- What are the characteristics that you bring to a friendship?
- What are the characteristics that you want in a friendship?
- What are the characteristics you avoid in a friendship?
- Describe any friendships you have made from doing group activities.
- Describe why these friendships formed.
- Describe how the friendships you may have had at the beginning of the year (e.g., August) have changed between then and now. In what ways have group activities impacted your friendships?
- What are the differences between friendships you make doing classroom group activities and other friendships you may have?
- Describe your closest friendships and how you got to know these individuals.
- Describe your relationships with peers in your classes. Are there other students that 1) you like to talk to, 2) you share common interests with, 3) you share a mutual understanding with, 4) you argue with but you always resolve the argument without hurting the relationships, 5) you think are caring, 6) you respect, 7) you can trust or are loyal, 8) have the same friends as you, 9) are friendly in the classroom but you do not hang out outside of class.
- How do you make new friends? What do you do, or what strategies do you have, to make new friends?
- How do group activities make it easier or harder for you to make or maintain friendships? Do group activities affect how you talk to or interact with other students? Can group activities help you find common interests with other students?
- Describe your interactions with other students during group activities: respectful, constructive, mutually encouraging, combative, argumentative, smooth, bumpy.
- How do your interactions during group activities compare to how you interact with the same peers online?
- How does your teacher affect how group activities go? How does your teacher influence how you make friends in the classroom?

#### Domain 3: Effects of collaborative learning on mental health and health behaviors.

Some people say that our friends influence our likes, dislikes, habits, behaviors, emotions and self-esteem. In your experience, how do your friends affect you? How do your classmates affect you?

**Probes:**

- Think of the friendships you have made or strengthened in the classroom. Describe how those individuals have influenced you. How have they influenced your health? Your mental health? Your behaviors?
- How do you think friends affect other kids you know?
- How do friends influence the likelihood that someone will get into trouble like fighting, bullying, skipping class, or using drugs?
- How have the friends of your friends influenced you?
- Think of 3 things that worry you the most. Which of those things are related to your relationships with friends?

#### WRAP UP.

I am going to summarize what I learned and I want you to let me know if I got anything wrong or if we left anything out.

(summarize discussion)

**Probes:**

- Did I get anything wrong? Did I miss anything? Anything else you think I should know?
- What other group activities do you do when you work with peers toward a common goal? Sports? Clubs?
